# Identify potential clinical significance of long noncoding RNA forkhead box P4 antisense RNA 1 in patients with early stage pancreatic ductal adenocarcinoma

**DOI:** 10.1002/cam4.2818

**Published:** 2020-01-28

**Authors:** Xiao‐Guang Liu, Hao Xu, Ming Chen, Xiao‐Yu Tan, Xiao‐Feng Chen, Yong‐Guang Yang, Man‐Zhou Lin, Guo‐Hua Liu, Xiao‐Lu Liang, Yi‐Bin Qian, Guo‐Jia Yuan, Min‐Qiang Chen, Wen‐Tao Li, Hui‐Lai Miao, Ming‐Yi Li, Xi‐Wen Liao, Wei Dai, Nian‐Ping Chen

**Affiliations:** ^1^ Department of Hepatobiliary Surgery Affiliated Hospital of Guangdong Medical University Zhanjiang Guangdong Province People's Republic of China; ^2^ Department of Hepatobiliary Surgery The First Affiliated Hospital of Guangxi Medical University Nanning Guangxi Zhuang Autonomous Region People's Republic of China

**Keywords:** bioinformatics, clinical significance, FOXP4‐AS1, pancreatic ductal adenocarcinoma, The Cancer Genome Atlas

## Abstract

Previous studies have shown that forkhead box P4 antisense RNA 1 (FOXP4‐AS1) is dysregulated in tumor tissues and can serve as a prognostic indicator for multiple cancers. However, the clinical significance of FOXP4‐AS1 in pancreatic ductal adenocarcinoma (PDAC) remains unclear. The goal of this study is to recognize the possible clinical significance of long noncoding RNA FOXP4‐AS1 in patients with early stage PDAC. A total of 112 patients from The Cancer Genome Atlas (TCGA) PDAC cohort, receiving RNA sequencing, were involved in the study. Survival analysis, functional mechanism, and potential small molecule drugs of target therapy of FOXP4‐AS1 were performed in this study. Survival analysis in TCGA PDAC cohort suggested that patients with high FOXP4‐AS1 expression had significantly augmented possibility of death than in PDAC patients with lower FOXP4‐AS1 expression (adjusted *P* = .008; adjusted HR = 2.143, 95% CI = 1.221‐3.760). In this study, a genome‐wide RNA sequencing dataset was used to identify 927 genes co‐expressing with FOXP4‐AS1 in PDAC tumor tissues. A total of 676 differentially expressed genes were identified between different FOXP4‐AS1 expression groups. Functional enrichment analysis of these genes and gene set enrichment analysis for PDAC genome‐wide RNA sequencing dataset was done. We have found that FOXP4‐AS1 may function in PDAC by participating in biological processes and pathways including oxidative phosphorylation, tricarboxylic acid cycle, classical tumor‐related pathways such as NF‐kappaB as well as Janus kinase/signal transducers in addition to activators of transcription, cell proliferation, and adhesion. In addition, we also screened two potential targeted therapeutic small molecule drugs (dimenhydrinate and metanephrine) for FOXP4‐AS1 in PDAC. In conclusion, our present study demonstrated that higher expression of FOXP4‐AS1 in PDAC tumor tissues were related with an inferior medical outcome. Through multiple genome‐wide approaches, we identified the potential molecular mechanisms of FOXP4‐AS1 in PDAC and two targeted therapeutic drugs for it.

## Graphical abstract

Survival analysis between high‐ and low‐forkhead box P4 antisense RNA 1 (FOXP4‐AS1) expression groups in pancreatic ductal adenocarcinoma (PDAC) overall survival (OS). (A) Expression of FOXP4‐AS1and PDAC patients' survival time scatter gram; (B) the survival curves between high‐ and low‐FOXP4‐AS1 expression groups; (C) time‐dependent ROC curve of FOXP4‐AS1 expression in predicting PDAC OS.

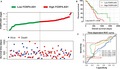

## 1 INTRODUCTION

Pancreatic cancer is an extremely malignant tumor with a low survival frequency. The majority of pancreatic cancer histology includes pancreatic ductal adenocarcinoma (PDAC). More and more literature reports that long noncoding RNA (lncRNA) has a crucial role in tumors, by means of participating in tumorigenesis, progression, tumor metastasis as well as prognosis.^1^, ^2^, ^3^, ^4^ Nowadays, a large number of high‐throughput sequencing datasets are shared in public databases for researchers to have open access to downloads, especially The Cancer Genome Atlas (TCGA) database from National Cancer Institute as well as National Human Genome Research Institute.^5^ Previous studies have used TCGA data to identify pancreatic cancer‐related lncRNA molecular markers and to screen a number of lncRNAs associated with pancreatic cancer prognosis.^6^, ^7^ However, since TCGA database contains a large amount of genome‐wide RNA sequencing dataset, and previous studies have failed to fully analyze these data. Therefore, the lncRNA markers related to the prognosis of pancreatic cancer from TCGA cohort need to be further explored. Previous studies have reported that lncRNA forkhead box P4 antisense RNA 1 (FOXP4‐AS1) is suggestively up‐regulated in colorectal cancer (CRC) tumor tissues; besides higher expression of FOXP4‐AS1 is correlated with poor outcome in CRC.^8^ Similar results can be observed in prostate cancer (PCa) and osteosarcoma.^9^, ^10^ However, the clinical significance of FOXP4‐AS1 in various cancers remains unclear, including pancreatic cancer. The goal of this study is to identify the potential clinical significance of lncRNA FOXP4‐AS1 in patients with early stage PDAC, including the prognosis value, functional mechanism, and potential small molecule drugs of target therapy.

## 2 MATERIALS AND METHODS

### 2.1 Data acquisition

The level 3 raw RNA sequencing dataset (including mRNAs and lncRNAs) of PDAC were acquired from the TCGA data portal (https://portal.gdc.cancer.gov/).^11^ University of California, Santa Cruz Xena provided complete clinical parameters of TCGA PDAC cohort. Patient inclusion and exclusion criteria were similar to our previously published manuscripts.^12^, ^13^, ^14^ We only included patients with early stage PDAC who experienced pancreaticoduodenectomy from the TCGA cohort. The patients who did not receive pancreaticoduodenectomy treatment or had an advance stage of the condition (stage III or IV), or without RNA sequencing dataset were excluded from this study. All datasets in this study were downloaded and used in accordance with TCGA's publication guidelines. Raw RNA sequencing dataset was normalized via DESeq bioconductor package in the R platform.^15^

### 2.2 Survival analysis of FOXP4‐AS1 in early stage PDAC

In this study, high‐ and low‐expression groups were cut off by the median expression value of FOXP4‐AS1. Prognostic differences between different FOXP4‐AS1 expression groups were compared using Kaplan‐Meier with log‐rank test, univariate as well as multivariate Cox proportional hazard regression models. Time‐dependent receiver operating characteristic (ROC) curve was used for evaluating the accuracy of FOXP4‐AS1 expression in predicting the prognosis of PDAC, which was performed by *survival* package in the R platform.^12^, ^16^ The nomogram was used to assess the contribution of FOXP4‐AS1 expression to PDAC prognosis after receiving pancreaticoduodenectomy treatment, which was performed by *rms* package in the R platform. Joint effects survival analysis was used to assess the predictive value of FOXP4‐AS1 expression and clinical parameters combination for PDAC prognosis.

### 2.3 Functional enrichment of FOXP4‐AS1 co‐expression genes

It is well known that lncRNA belongs to noncoding RNA and is involved in the regulation of protein coding genes (PCGs) mRNA expression levels in the post‐transcriptional phase. The function of lncRNA is mainly through the regulation of the expression level of PCGs. Therefore, there is a relationship between the specific lncRNA‐regulated PCGs and the corresponding lncRNA. In this study, we used the Pearson correlation coefficient (*r*) to screen and identify FOXP4‐AS1‐related PCGs. Protein coding genes with a *P* < .05 and |*r*| > .2 were identified as FOXP4‐AS1‐related PCGs.^17^, ^18^ Functional evaluation of these FOXP4‐AS1‐related PCGs was done by means of the Database for Annotation, Visualization, and Integrated Discovery (DAVID) v6.8.^19^, ^20^ Biological Networks Gene Ontology tool (BiNGO)^21^ was used to reveal the biological function of FOXP4‐AS1 in PDAC tumor tissues. Interaction relationship of these FOXP4‐AS1‐related PCGs was revealed by Gene Multiple Association Network Integration Algorithm (GeneMANIA; http://genemania.org/)^22^, ^23^ and the Search Tool for the Retrieval of Interacting Genes/Proteins (STRING).^24^, ^25^, ^26^

### 2.4 Differentially expressed genes screening and functional annotation

In order to reveal the mechanisms involved in the prognosis between different FOXP4‐AS1 expression levels, we used the *edgeR* bioconductor package to screen differentially expressed genes (DEGs) between different FOXP4‐AS1 expression groups of PDAC patients' tumor tissues.^27^ Genes with the |log_2_ fold change| ≥ 1, the *P* value and false discovery rate (FDR) < 0.05 were identified as DEGs among low as well as high FOXP4‐AS1 expression groups in PDAC tumor tissues.^17^ Database for Annotation, Visualization, and Integrated Discovery v6.8 and BiNGO were used to identify the biological function and pathway between high and low FOXP4‐AS1expression groups. The interaction relationship of these DEGs was revealed by GeneMANIA and STRING. The Connectivity Map (CMap) was used to identify potential small molecule targeted drugs for FOXP4‐AS1 in PDAC.^28^, ^29^ Those with small molecule DRUGS a mean connective score <−0.2 and *P* < .05 were recognized as the possible therapeutic drugs of FOXP4‐AS1 in PDAC.

### 2.5 Gene set enrichment analysis of PDAC whole‐genome dataset and FOXP4‐AS1 expression levels

To further examine the potential mechanism between diverse FOXP4‐AS1 expression groups in PDAC tumor tissues, we performed a Gene set enrichment analysis (GSEA) using c2 and c5 Molecular Signatures Database gene sets as the reference gene set.^30^, ^31^, ^32^ Results with a nominal *P* < .05, |normalized enrichment score| > 1 and FDR < 0.25 were regarded as statistically significant.

### 2.6 Statistical analysis

For multivariate Cox proportional hazard regression model, those clinical parameters with a log‐rank *P* < .05 were fitted into the model for adjustment. False discovery rate in GSEA and DEGs screening were performed according to the Benjamini‐Hochberg procedure.^33^ Volcano plots as well as heat maps were created via the *ggplot2* package in the R platform. A *P* < .05 was regarded statistically significant. Biological Networks Gene Ontology analysis was performed in the cytoscape v3.6.1. Data analysis was done by SPSS version 22.0 (IBM Corporation USA) and R 3.4.4.

## 3 RESULTS

### 3.1 Data preprocessing

The raw count RNA sequencing dataset was normalized by means of DESeq package, and a total of 112 early stage PDAC patients who experienced pancreaticoduodenectomy were incorporated into this study. The demographic epidemiological data of PDAC patients have been described in our previous studies, and also shown in Table S1. In clinical parameters, we observed that histological score, radiation treatment, radical resection as well as targeted molecular treatment were suggestively related with PDAC overall survival (OS) in this study, and were required to be fitted into the multivariate Cox proportional hazard regression model as adjusted variables.

### 3.2 Survival analysis of FOXP4‐AS1 in early stage PDAC

**Figure 1:**
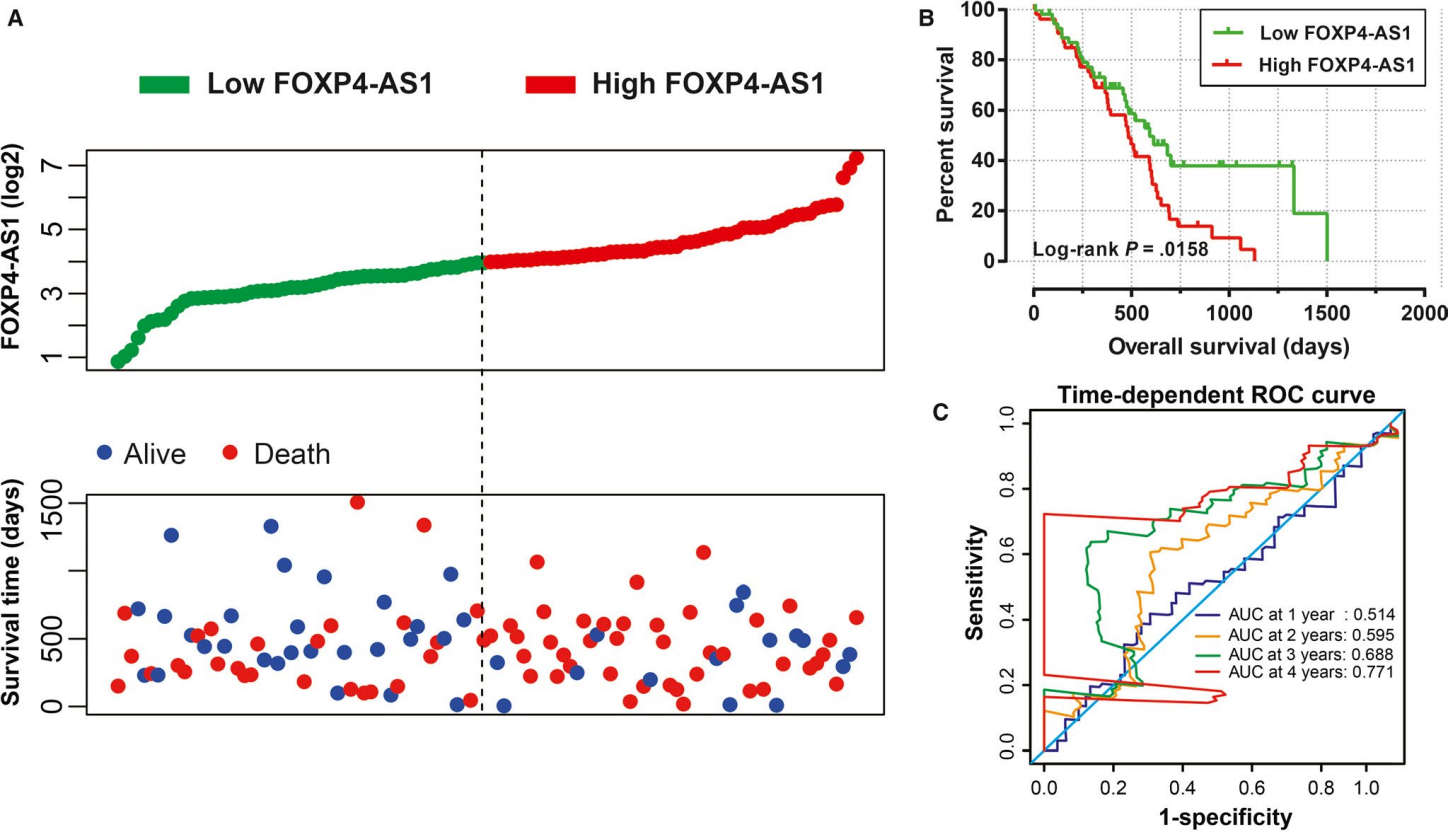
Survival analysis between high‐ and low‐forkhead box P4 antisense RNA 1 (FOXP4‐AS1) expression groups in pancreatic ductal adenocarcinoma (PDAC) overall survival (OS). A, Expression of FOXP4‐AS1and PDAC patients' survival time scatter gram; (B) the survival curves between high‐ and low‐FOXP4‐AS1 expression groups; (C) time‐dependent receiver operating characteristic (ROC) curve of FOXP4‐AS1 expression in predicting PDAC OS

**Figure 2:**
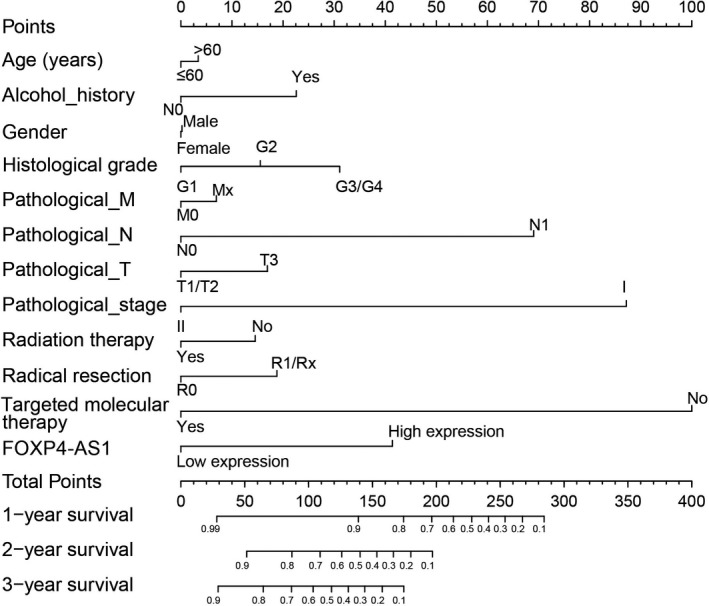
Nomogram between forkhead box P4 antisense RNA 1 (FOXP4‐AS1) and clinical parameters in The Cancer Genome Atlas (TCGA) pancreatic ductal adenocarcinoma (PDAC) cohort

**Figure 3:**
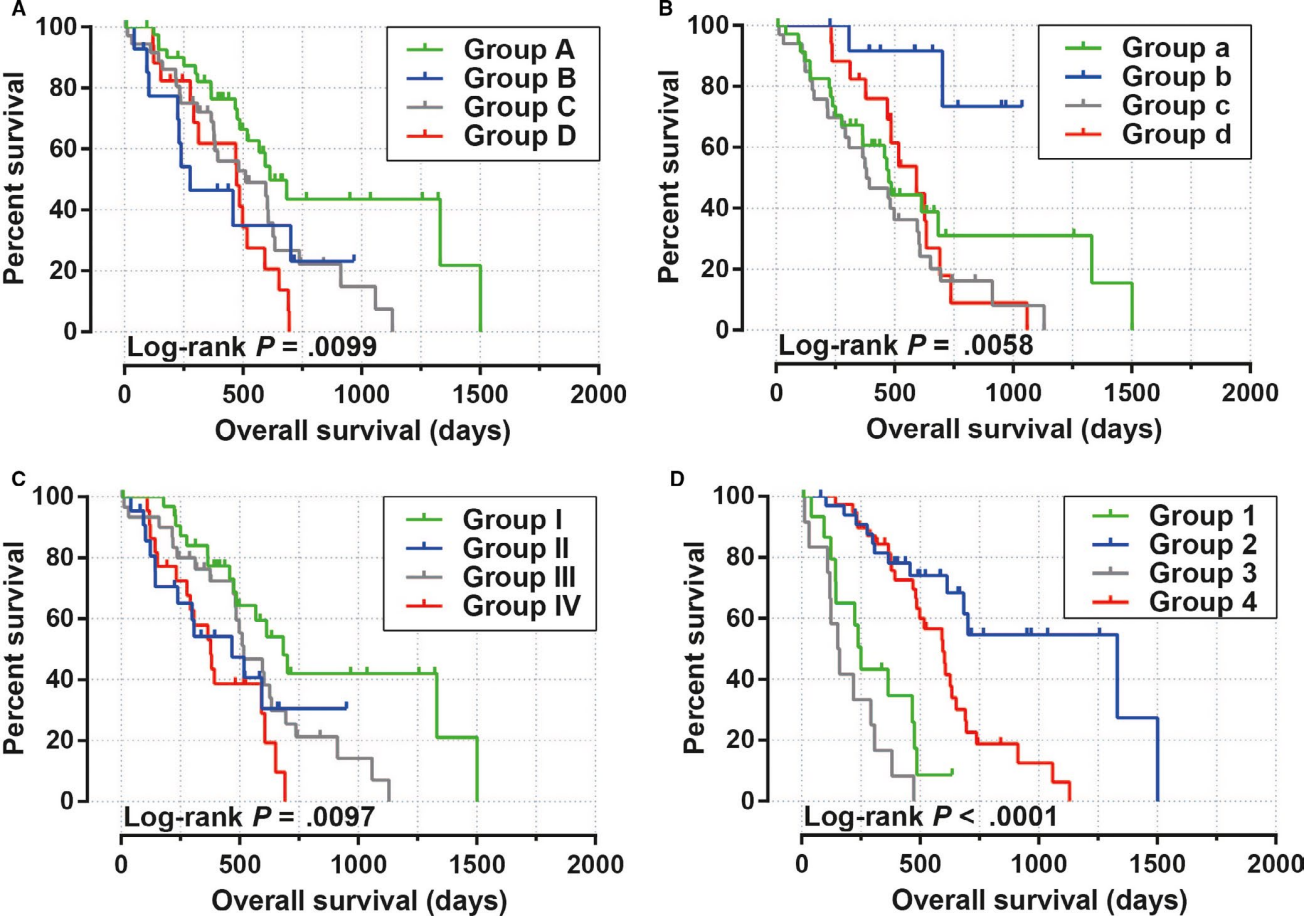
Joint effect survival analysis between forkhead box P4 antisense RNA 1 (FOXP4‐AS1) and clinical parameters in The Cancer Genome Atlas (TCGA) pancreatic ductal adenocarcinoma (PDAC) cohort; (A) FOXP4‐AS1 combined with histological Grade; (B) FOXP4‐AS1 combined with radiation therapy; (C) FOXP4‐AS1 combined with radical resection; (D) FOXP4‐AS1 combined with targeted molecular therapy

Survival analysis was performed in different FOXP4‐AS1 expression groups. We observed that the high FOXP4‐AS1 expression was significantly associated with an unfavorable prognosis in PDAC, and increased risk of cancer‐related death of PDAC patients (adjusted *P* = .008; adjusted HR = 2.143; 95% CI = 1.221‐3.760). Patients with high FOXP4‐AS1 expression have a shorter median survival time compared with patients with low FOXP4‐AS1 expression (Log‐rank *P* = .0158, high FOXP4‐AS1 vs low FOXP4‐AS1 = 485 vs 593 days; Figure 1A,B). Time‐dependent ROC curve indicated that the expression level of FOXP4‐AS1 gene has a certain prognostic value in the long‐term survival of patients with PDAC. The area under the curve of ROC curve in 1‐, 2‐, 3‐, and 4‐year survival were 0.514, 0.595, 0.688, and 0.771, respectively (Figure 1C). The nomogram also suggested that FOXP4‐AS1 gene expression levels had a greater impact on the prognosis of patients with PDAC, and it ranked second to the TNM staging system and targeted molecular therapy (Figure 2). Joint effect survival analysis suggested that FOXP4‐AS1 combined with clinical parameters could improve prognostic prediction performance (Figure 3A‐D). Multivariate Cox proportional hazard regression model of joint effect survival analysis also supported this conclusion except for the combination with radiation therapy (Table 1).

**Table 1:** Joint effects survival analysis of clinical factors and the FOXP4‐AS1 expression with OS in PDAC patients

Group	FOXP4‐AS1	Variables	Patients (n = 112)	MST (d)	Crude HR (95% CI)	Crude *P*	Adjusted HR (95% CI)	Adjusted *P* a
		Histological grade						
A	Low expression	G1 + G2	42	614	1		1	
B	Low expression	G3 + G4	14	277	2.460 (1.087‐5.571)	.031	4.758 (1.921‐11.783)	.001
C	High expression	G1 + G2	38	511	2.029 (1.097‐3.750)	.024	3.606 (1.755‐7.408)	.0005
D	High expression	G3 + G4	18	473	3.035 (1.497‐6.152)	.002	4.035 (1.818‐8.954)	.001
		Radiation therapy^b^						
a	Low expression	No	36	476	1		1	
b	Low expression	Yes	13	NA	0.181 (0.042‐0.781)	.022	0.313 (0.068‐1.439)	.136
c	High expression	No	34	381	1.564 (0.867‐2.822)	.137	1.682 (0.899‐3.148)	.104
d	High expression	Yes	17	592	1.137 (0.559‐2.314)	.723	1.840 (0.785‐4.314)	.161
		Radical resection^c^						
I	Low expression	R0	33	684	1		1	
II	Low expression	R1/Rx	22	467	2.406 (1.096‐5.285)	.029	2.490 (1.012‐6.123)	.047
III	High expression	R0	33	517	2.011 (1.030‐3.927)	.041	2.796 (1.360‐5.749)	.005
IV	High expression	R1/Rx	22	378	3.316 (1.594‐6.895)	.001	3.542 (1.618‐7.755)	.002
		Targeted molecular therapy^d^						
1	Low expression	No	16	250	1		1	
2	Low expression	Yes	34	1332	0.121 (0.051‐0.288)	<.0001	0.095 (0.036‐0.254)	<.0001
3	High expression	No	13	153	2.167 (0.959‐4.895)	.063	1.484 (0.630‐3.495)	.366
4	High expression	Yes	39	596	0.311 (0.153‐0.631)	.001	0.268 (0.122‐0.589)	.001

Abbreviations: CI, confidence interval; HR, hazard ratio; MST, median survival time; OS, overall survival; PDAC, pancreatic ductal adenocarcinoma.

a Adjusted for histological grade, radiation therapy, radical resection and targeted molecular therapy.

b Radiation therapy information are unavailable in 12 patients.

c Radical resection information are unavailable in 2 patients.

d Targeted molecular therapy information are unavailable in 10 patients.

### 3.3 Functional enrichment of FOXP4‐AS1 co‐expression genes

**Figure 4:**
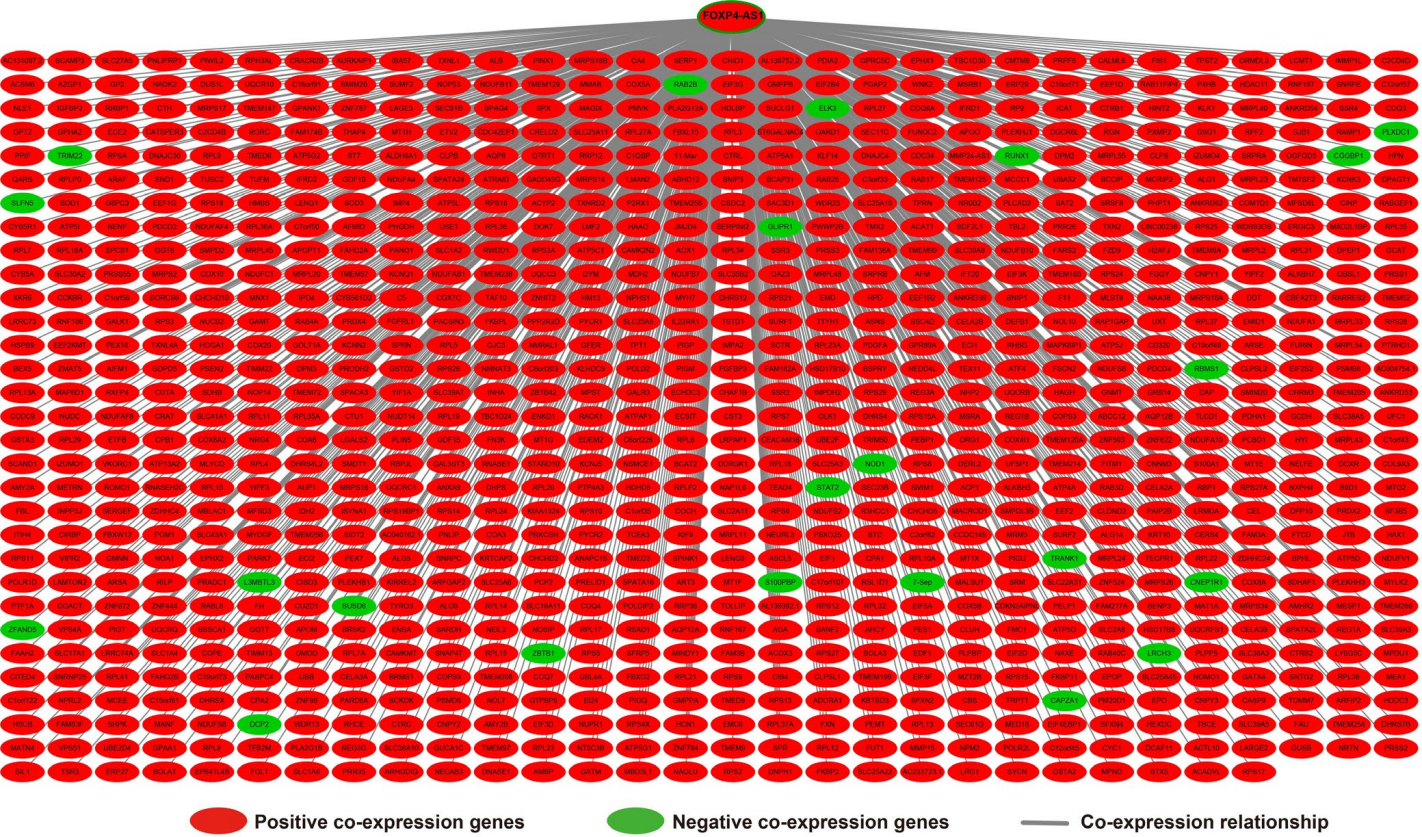
Co‐expression of regulatory networks between forkhead box P4 antisense RNA 1 and its co‐expression protein coding genes

**Figure 5:**
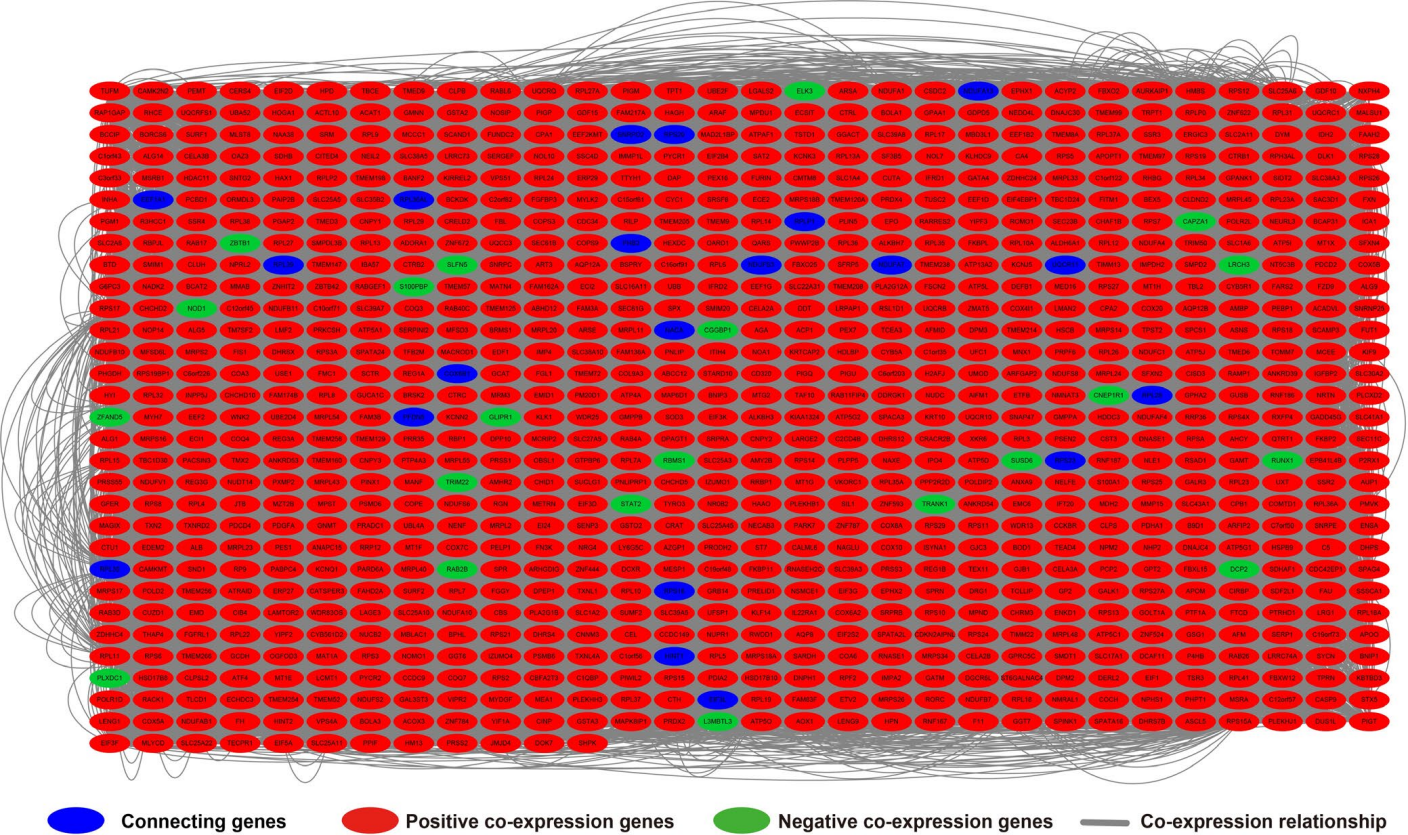
Co‐expression of regulatory networks of forkhead box P4 antisense RNA 1 co‐expression protein coding genes that constructed by GeneMANIA

**Figure 6:**
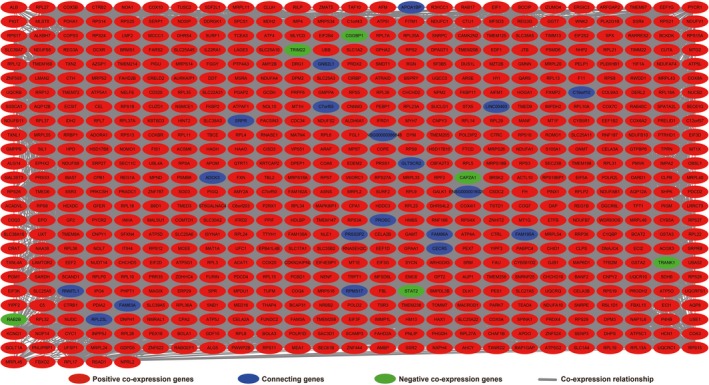
Co‐expression of regulatory networks of forkhead box P4 antisense RNA 1 co‐expression protein coding genes that constructed by STRING

Furthermore, we conducted a genome‐wide co‐expression analysis of FOXP4‐AS1 to screen 927 FOXP4‐AS1‐related PCGs. Out of these 927 PCGs, 22 PCGs were negatively correlated with FOXP4‐AS1, whereas 905 PCGs were positively correlated with FOXP4‐AS1 (Figure 4; Table S2). Then, we used DAVID v6.8 to evaluate the function of these genes and found that these FOXP4‐AS1‐related PCGs were significantly associated with cell‐cell adhesion, focal adhesion, cell‐cell adherence junction, tricarboxylic acid (TCA) cycle, deleterious regulation of growth, regulation of necrotic cell death, DNA impairment response, detection of DNA damage, and lipid catabolic process by Gene Ontology (GO) analysis (Table S3). Kyoto Encyclopedia of Genes and Genomes (KEGG) analysis revealed that these PCGs were expressively associated with oxidative phosphorylation, pancreatic secretion, glyoxylate and dicarboxylate metabolism, peroxisome, ubiquinone, and other terpenoid‐quinone biosynthesis, and the Citrate cycle (Table S4). Functional evaluation by BiNGO also demonstrated that these PCGs were significantly associated with ncRNA metabolic process, ncRNA processing, mRNA binding, oxidoreductase activity, NADH or NADPH, and oxidative phosphorylation (Figure S1). The gene‐gene interaction and protein‐protein interaction (PPI) networks that were constructed by GeneMANIA and STRING online tools are also shown in Figures 5 and 6, respectively. We observed that these genes have complex interaction networks and are co‐expressed with each other.

### 3.4 DEGs screening and functional annotation

**Figure 7:**
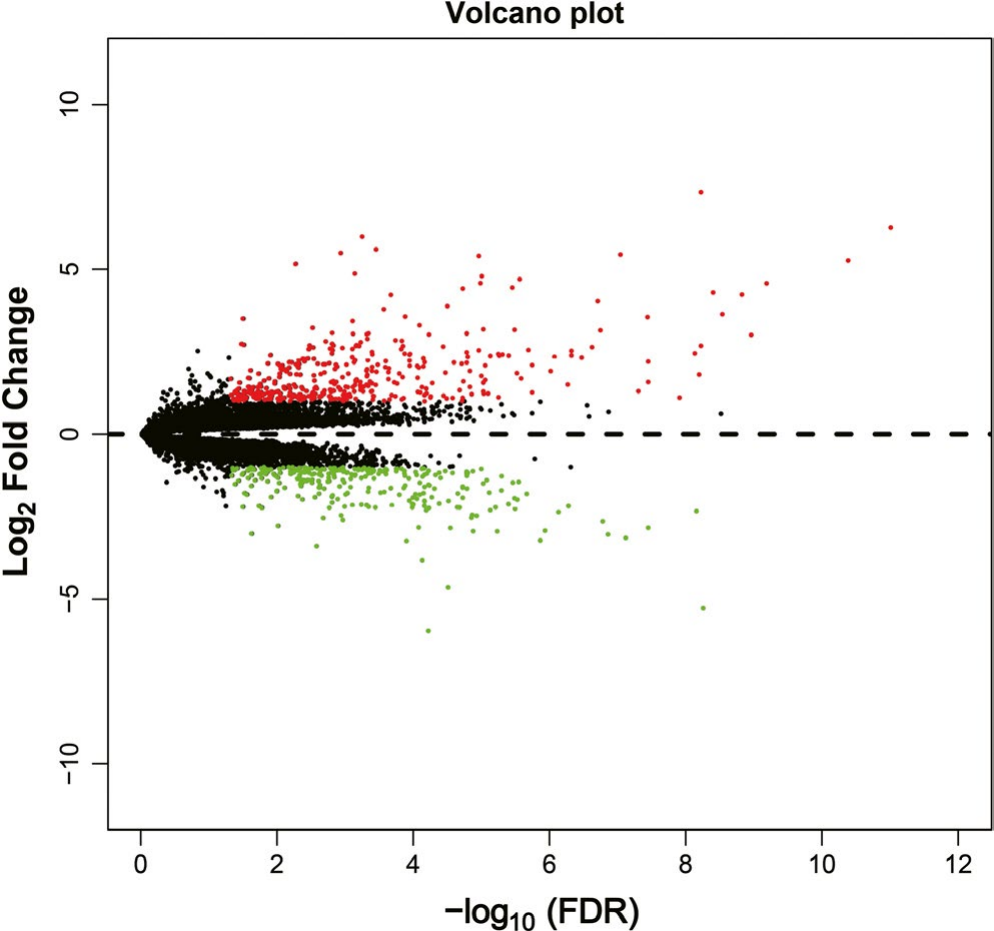
Volcano plot of differentially expressed genes between high‐ and low‐ forkhead box P4 antisense RNA 1 expression groups in pancreatic ductal adenocarcinoma tumor tissues

**Figure 8:**
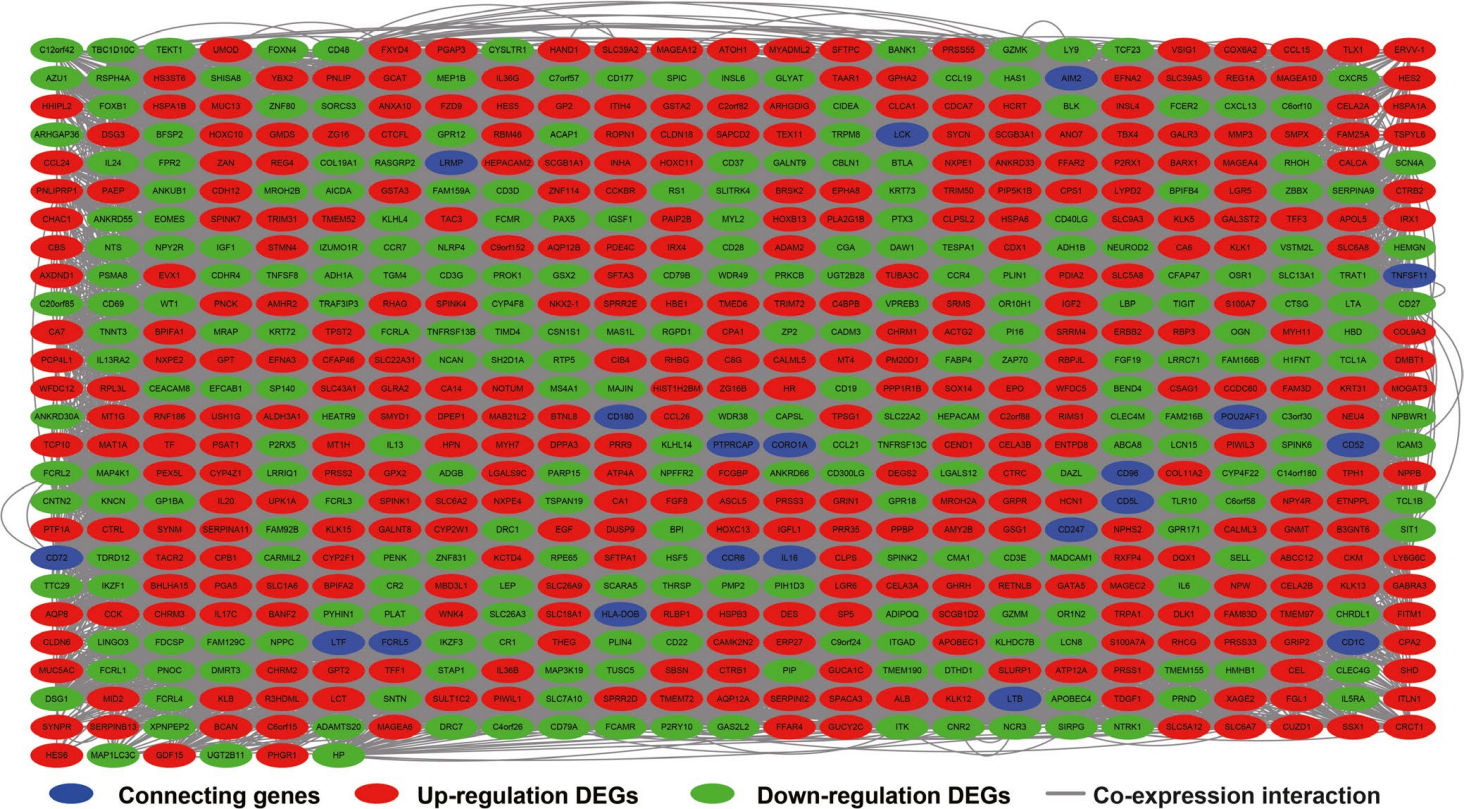
Co‐expression of regulatory networks of differentially expressed genes between high‐ and low‐forkhead box P4 antisense RNA 1 expression groups in pancreatic ductal adenocarcinoma tumor tissues that was constructed by GeneMANIA

**Figure 9:**
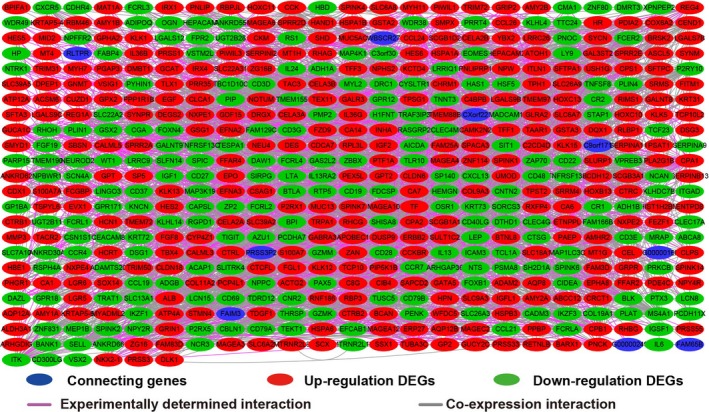
Regulatory networks of differentially expressed genes between high‐ and low‐forkhead box P4 antisense RNA 1 expression groups in pancreatic ductal adenocarcinoma tumor tissues that was constructed by STRING

**Figure 10:**
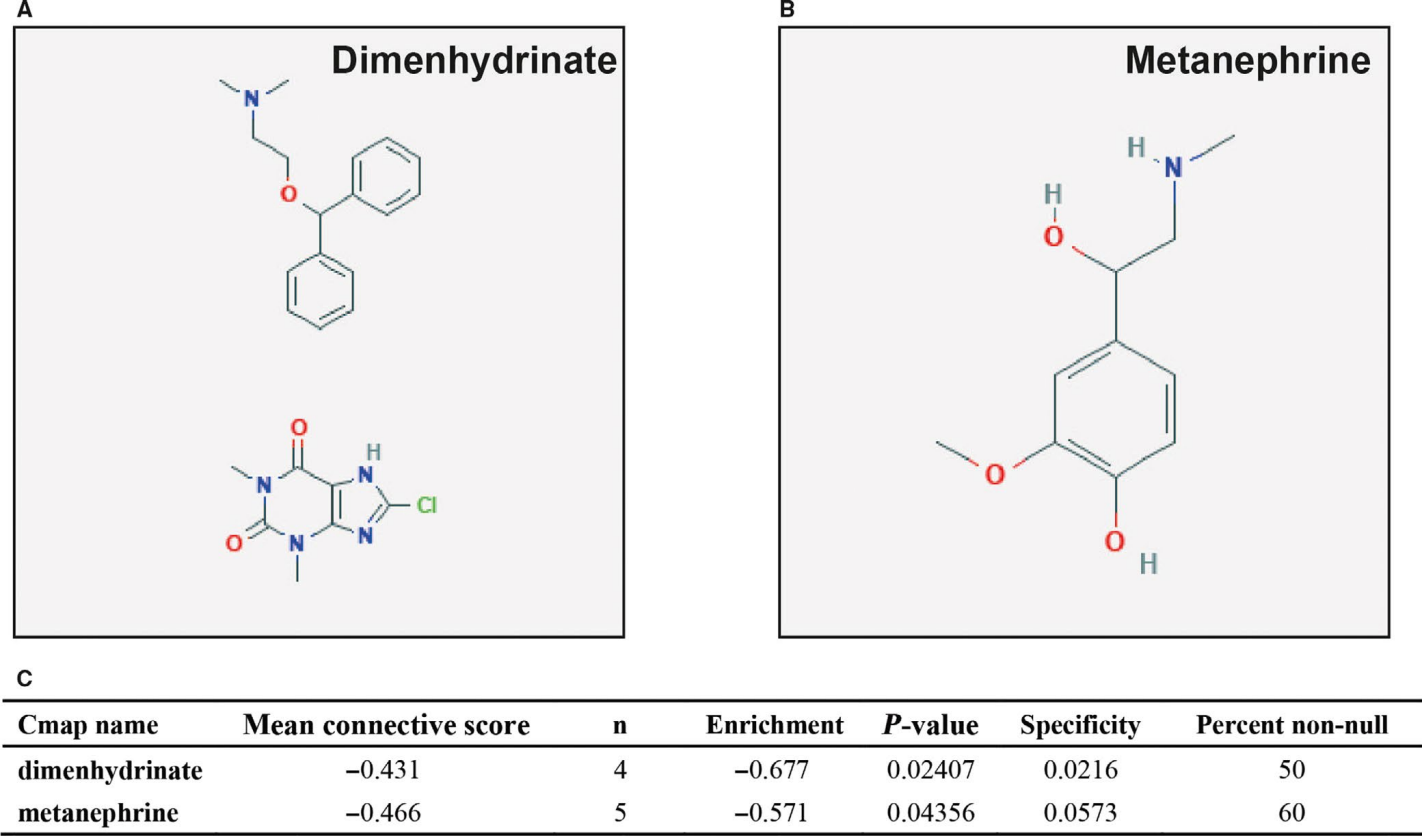
Connectivity Map (CMap) analysis results; (A) chemical structure of dimenhydrinate; (B) chemical structure of metanephrine; (C) CMap analysis results

Based on the median value of FOXP4‐AS1 expression, we grouped and screened DEGs between low and high FOXP4‐AS1 expression groups. A total of 676 DEGs were obtained. Out of these, 285 were down‐regulated and 391 were up‐regulated (Table S5). Volcano plot and heat map of DEGs are shown in Figure 7 and Figure S2, respectively. Functional assessment of these DEGs was evaluated by DAVID v6.8. Gene Ontology term analysis revealed that these DEGs was suggestively associated with cell surface receptor signaling cascade, chemokine‐mediated signaling cascade, regulation of phosphatidylinositol 3‐kinase signaling, positive regulation of mitotic nuclear division, regulation of angiogenesis, and cell propagation, positive regulation of Janus kinase/signal transducers as well as stimulators of transcription (JAK/STAT) cascade (Table S6). Kyoto Encyclopedia of Genes and Genomes analysis also suggested that these DEGs were significantly associated with pancreatic secretion, cytokine‐cytokine receptor interaction, calcium signaling cascade, metabolism of xenobiotics by cytochrome P450, cell adhesion molecules, and drug metabolism—cytochrome P450 (Table S7). Biological Networks Gene Ontology analysis also showed that these DEGs were significantly associated with cell differentiation, regulation of cell proliferation, cell adhesion, regulation of tumor necrosis factor production, and positive regulation of MAPKKK cascade (Figure S3). Gene‐gene interaction and PPI networks that were constructed by GeneMANIA and STRING online tools are also shown in Figures 8 and 9, respectively. Gene‐gene interaction and PPI networks revealed that these DEGs were complex and were co‐expressed with each other. Subsequently, we performed a CMap analysis to identify two small molecule drugs that could be used as potential targeted therapeutic drugs for FOXP4‐AS1 in PDAC. The chemical structures of these two small molecule drugs are shown in Figure 10A,B. They are dimenhydrinate (Mean connective score = −0.431; *P* = .02407; Figure 10C) and metanephrine (mean connective score = −0.466; *P* = .04356; Figure 10C).

### 3.5 GSEA of PDAC whole‐genome dataset and FOXP4‐AS1 expression levels

**Figure 11:**
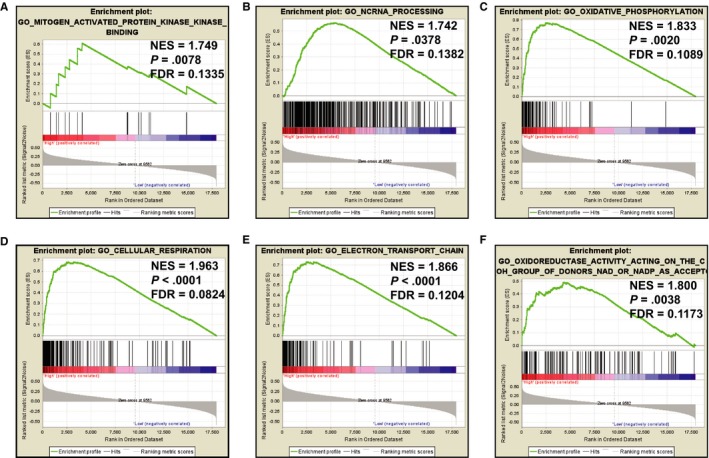
Gene set enrichment analysis results enriched in phenotype of high‐forkhead box P4 antisense RNA 1 expression group using the c5 reference gene set (A) MITOGEN‐ACTIVATED PROTEIN KINASE KINASE BINDING; (B) NCRNA PROCESSSING; (C) OXIDATIVE PHOSPHORYLATION; (D) CELLULAR RESPIRATION; (E) ELECTRON TRANSPORT CHAIN; (F) OXIDOREDUCTASE ACTIVITY ACTING ON THE CH‐OH GROUP OF DONORS NAD OR NADP AS ACCEPTOR

**Figure 12:**
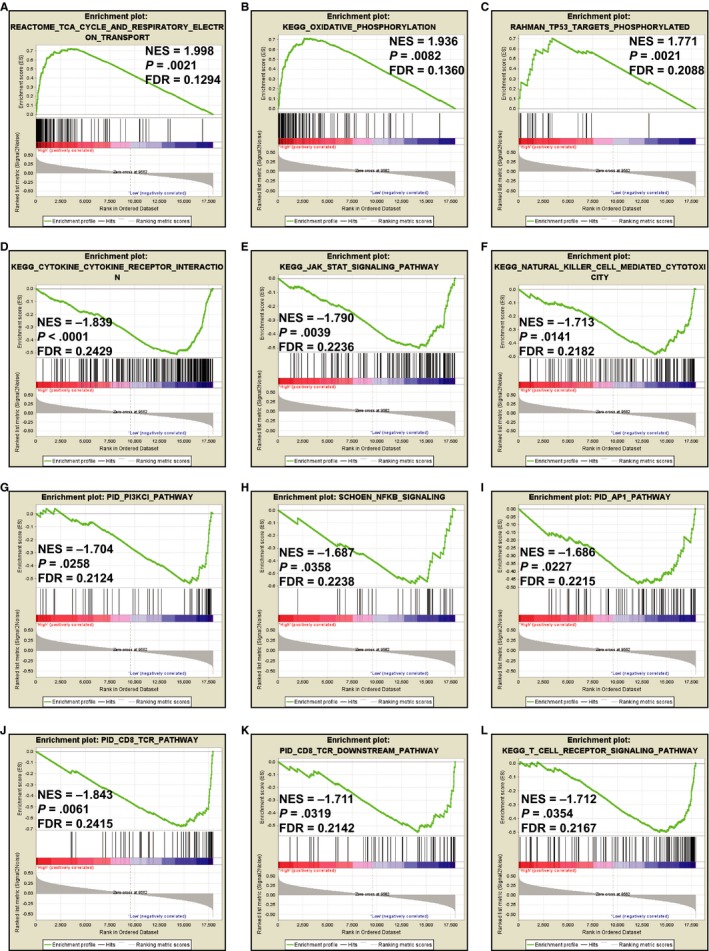
Gene set enrichment analysis results enriched in phenotype of high forkhead box P4 antisense RNA 1 expression group: (A) TCA CYCLE AND RESPIRATORY ELECTR ON TRANSPORT; (B) OXIDATIVE PHOSPHORYLATION; (C) TP53 TARGETS PHOSPHORYLATED. As well as low FOXP4‐AS1 expression group (D) CYTOKINE CYTOKINE RECEPTOR INTERACTION; (E) JAK STA SIGNALING PATHWAY; (F) NATURAL KILLER CELL MEDIATED CYTOTOXICITY; (G) PI3KCI PATHWAY; (H) NFKB SIGNALING; (I)API PATHWAY; (J) CD8 TCR PATHWAY; (K) CD8 TCR DOWNSTREAM PATHWAY; (L) T CELL RECEPTOR SIGNALING PATHWAY, by using the c5 reference gene set

To further understand the molecular mechanism of FOXP4‐AS1 in PDAC, we used the GSEA method to analyze whole‐genome dataset of PDAC tumor tissues between different FOXP4‐AS1 expression levels. GSEA analysis with c5 as a reference gene set revealed that biological processes of cellular respiration, electron transport chain, oxidative phosphorylation, oxidoreductase action on the CH‐OH group of donors, NAD or NADP as acceptors, Mitogen Activated Protein Kinase binding and ncRNA processing were significantly associated with high FOXP4‐AS1 expression PDAC (Figure 11A‐F; Table S8). However, there was no significant difference in low FOXP4‐AS1 expression PDAC group. GSEA analysis with c2 as a reference gene set found that cascades of TCA cycle and respiratory electron transport, oxidative phosphorylation, and TP53 targets were phosphorylated as well as were significantly associated with high FOXP4‐AS1 expression PDAC (Figure 12A‐C; Table S9). Whereas, pathways of CD8 T cell receptor (TCR), Class I phosphatidylinositol‐3 kinase, NF‐kappaB (NF‐kB), activator protein‐1, JAK/STAT, cytokine‐cytokine receptor interaction, natural killer cell‐mediated cytotoxicity, and TCR signaling cascade were significantly related with low FOXP4‐AS1 expression PDAC (Figure 12D‐L; Table S10).

## 4 DISCUSSION

Forkhead box P4 antisense RNA 1 is an emerging cancer‐related biomarker. Previously, there have been few reports on its relationship in tumors. After reviewing the literature, we found that preceding studies have documented the clinical values of FOXP4‐AS1 in PCa,^10^ CRC^8^ and osteosarcoma,^9^ as well as its biological function in cancers. Previously published studies have confirmed that FOXP4‐AS1 is significantly up‐regulated in CRC tumor tissues.^8^ Additionally, patients with high expression of FOXP4‐AS1 in CRC tumor tissues suggested an unfavorable prognosis. In addition, FOXP4‐AS1 expression is also related with the development of CRC.^8^ In vitro experimentations have shown that silencing of FOXP4‐AS1 in CRC cell lines can inhibit the cell proliferation and induce apoptosis. In vivo experiments confirmed that silencing of FOXP4‐AS1 in CRC cell lines can inhibit the tumor cells' ability to form tumors in nude mice.^8^ These data suggested that FOXP4‐AS1 might have an oncogene part in CRC and might assist as a biomarker for CRC progression and prognosis.^8^ Similar findings can also be found in osteosarcoma. Yang et al established that FOXP4‐AS1 is significantly up‐regulated in osteosarcoma tumor tissues, besides its high expression can significantly increase the danger of death and recurrence in patients with osteosarcoma. In vitro experiments confirmed that up‐regulation of FOXP4‐AS1 in osteosarcoma cell lines can promote osteosarcoma cell proliferation, invasion and migration, as well as cell cycle and inhibit apoptosis. The results of silencing the FOXP4‐AS1 in the osteosarcoma cell lines were contrary to the results of up‐regulating FOXP4‐AS1.^9^ Studies by Wu et al indicated that FOXP4‐AS1 is significantly up‐regulated in PCa tumor tissues and overexpressed in tumor tissues of PCa patients with advanced stage. Survival analysis found that patients with high expression of FOXP4‐AS1 had a poor outcome. In vivo and in vitro functional experiments suggest that FOXP4‐AS1 plays an oncogene role in PCa.^10^ In this study, we have reported that patients with higher manifestation of FOXP4‐AS1 in PDAC tumor tissues had significantly shorter OS times than patients with low FOXP4‐AS1. Our results are consistent with the results of previous studies.

Through genome‐wide co‐expression analysis, we found that FOXP4‐AS1 may participate in cell adhesion‐related biological processes, regulation of growth, and necrotic cell death, DNA damage‐related biological processes. It is well known that cell adhesion‐related molecules and biological processes play important roles in tumor metastasis,^34^ including pancreatic cancer.^35^ The necrotic cell death and DNA damage‐related biological process were the cell basic status, and may have a certain impact on tumor cell proliferation. By functional enrichment of DEGs in PDAC patients with different FOXP4‐AS1 expression levels, we found that these DEGs are also involved in cell adhesion‐related biological processes and proliferation, as well as cell cycle biological process, angiogenesis, JAK/STAT, and MAPKKK cascade. Studies have shown that cell cycle can be used as targeted treatment of pancreatic cancer,^36^ plus cell cycle inhibitors can be used as a new treatment for advanced pancreatic cancer.^37^ Angiogenesis is crucial for cancer development, and prevention of angiogenesis can be an important means of controlling tumor development. Therefore, prevention of angiogenesis is a valuable treatment for cancer, such as pancreatic cancer.^38^, ^39^, ^40^ Numerous studies have shown that specific molecules can affect the tumorigenesis, progression, incursion as well as migration of pancreatic cancer by participating in the regulation of JAK/STAT pathway.^41^, ^42^, ^43^, ^44^ MAPKKK cascade is the key molecule in the MAPK pathway, and MAPK pathway can be incorporated to control cell propagation, invasion, migration, apoptosis, tumorigenesis, and progression of pancreatic tumor, as well as chemotherapy response and clinical outcomes.^45^, ^46^, ^47^, ^48^, ^49^, ^50^, ^51^ For the two drugs that were identified in our current study, no previous studies have identified their functions on cancer treatment. These two drugs as the targeted agents for pancreatic cancer remain to be confirmed by further research as well as clinical trials.

The advantage of this study is that we have found for the first time that FOXP4‐AS1 can be used as a prognostic biomarker in patients with PDAC after pancreaticoduodenectomy. At the same time, we also used the joint effect survival analysis and nomogram to comprehensively analyze the prognostic values of FOXP4‐AS1 in patients with PDAC receiving pancreaticoduodenectomy. Furthermore, we also used the TCGA genome‐wide RNA sequencing dataset to screen the functional enrichment of FOXP4‐AS1 in PDAC tumor tissues by co‐expression and DEGs using GO and KEGG functional enrichment methods. The role of FOXP4‐AS1 in PDAC was further determined by GSEA enrichment.

Our study has some limitations that need to be explained. First, all the results were from a single cohort with small sample size. In the future, a large sample multicenter cohort is needed to verify our results. Second, the data from this study are derived from the TCGA database. There are some missing clinical parameters, which may have a certain impact on the quality of the research. Third, since the molecular mechanisms of this study are derived from functional annotation of genes and the GSEA approach, enrichment analysis results obtained in this study lack validation in vivo and in vitro experiments, as well as the effects of two potential small molecule drugs in PDAC. Despite the limitations, this study made full use of genome‐wide RNA sequencing dataset to comprehensively analyze the clinical significance, potential mechanism, and screening of targeted drugs of FOXP4‐AS1 in PDAC through multiple bioinformatics analysis approaches. This study is also the first to report the prognostic value of FOXP4‐AS1 in PDAC. It may provide a theoretical basis for the clinical application of FOXP4‐AS1 in PDAC.

## 5 CONCLUSION

In conclusion, our study has identified that high expression of FOXP4‐AS1 in PDAC tumor tissues were predicting an unfavorable prognosis. Through genome‐wide functional enrichment analysis, we found that FOXP4‐AS1 may be involved in the following biological processes and pathways in PDAC: oxidative phosphorylation, TCA cycle, tumor immunity, classical tumor‐related pathways such as NF‐kB and JAK/STAT, cell proliferation, and adhesion. In addition, we also screened two potential targeted therapeutic small molecule drugs (dimenhydrinate and metanephrine) for FOXP4‐AS1 in PDAC. However, our results are still to be further verified in the future.

## CONFLICT OF INTEREST

None declared.

## Data Availability

The datasets used during the present study are available from the corresponding author upon reasonable request. All raw data of PDAC, which include into current study, can be downloaded from TCGA (https://portal.gdc.cancer.gov/).
