# Supplementary figures and images for: Identify potential clinical significance of long noncoding RNA forkhead box P4 antisense RNA 1 in patients with early stage pancreatic ductal adenocarcinoma
Source: Cancer Med. 2020 Jan 28;9(6):2062–76. doi: 10.1002/cam4.2818 (PMC7064149; doi:10.1002/cam4.2818)

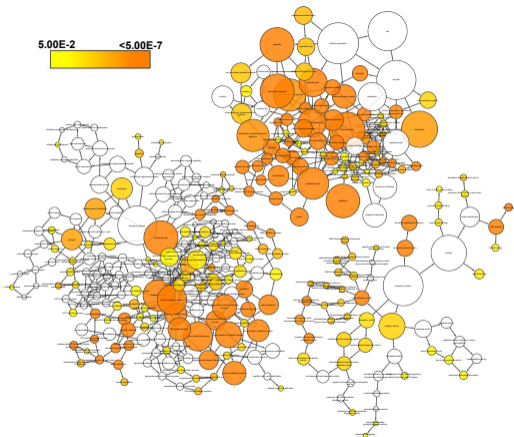

**Figure S1.** Directed acyclic graph of FOXP4-AS1-related PCGs using BiNGO analysis.

Supplement: Supplementary file 1 [file CAM4-9-2062-s001.pdf]

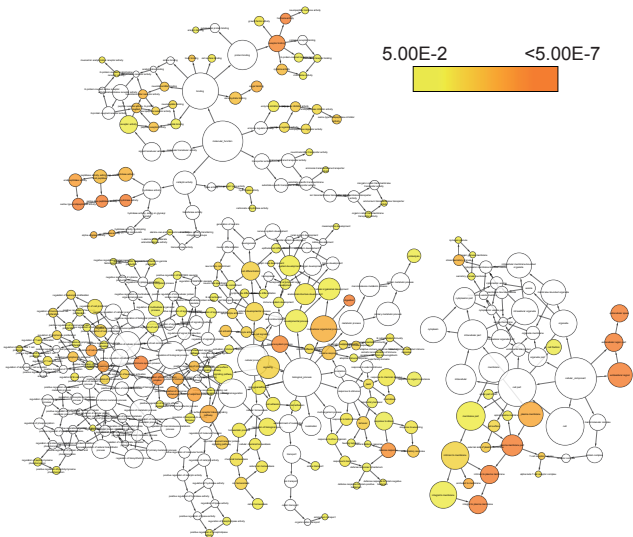

**Figure S3.** Directed acyclic graph of DEGs using BiNGO analysis.

Supplement: Supplementary file 3 [file CAM4-9-2062-s003.pdf]
