# Supplementary material for: Identify potential clinical significance of long noncoding RNA forkhead box P4 antisense RNA 1 in patients with early stage pancreatic ductal adenocarcinoma
Source: Cancer Med. 2020 Jan 28;9(6):2062–76. doi: 10.1002/cam4.2818 (PMC7064149; doi:10.1002/cam4.2818)
Supplement: Supplementary file 2 [file CAM4-9-2062-s002.pdf]

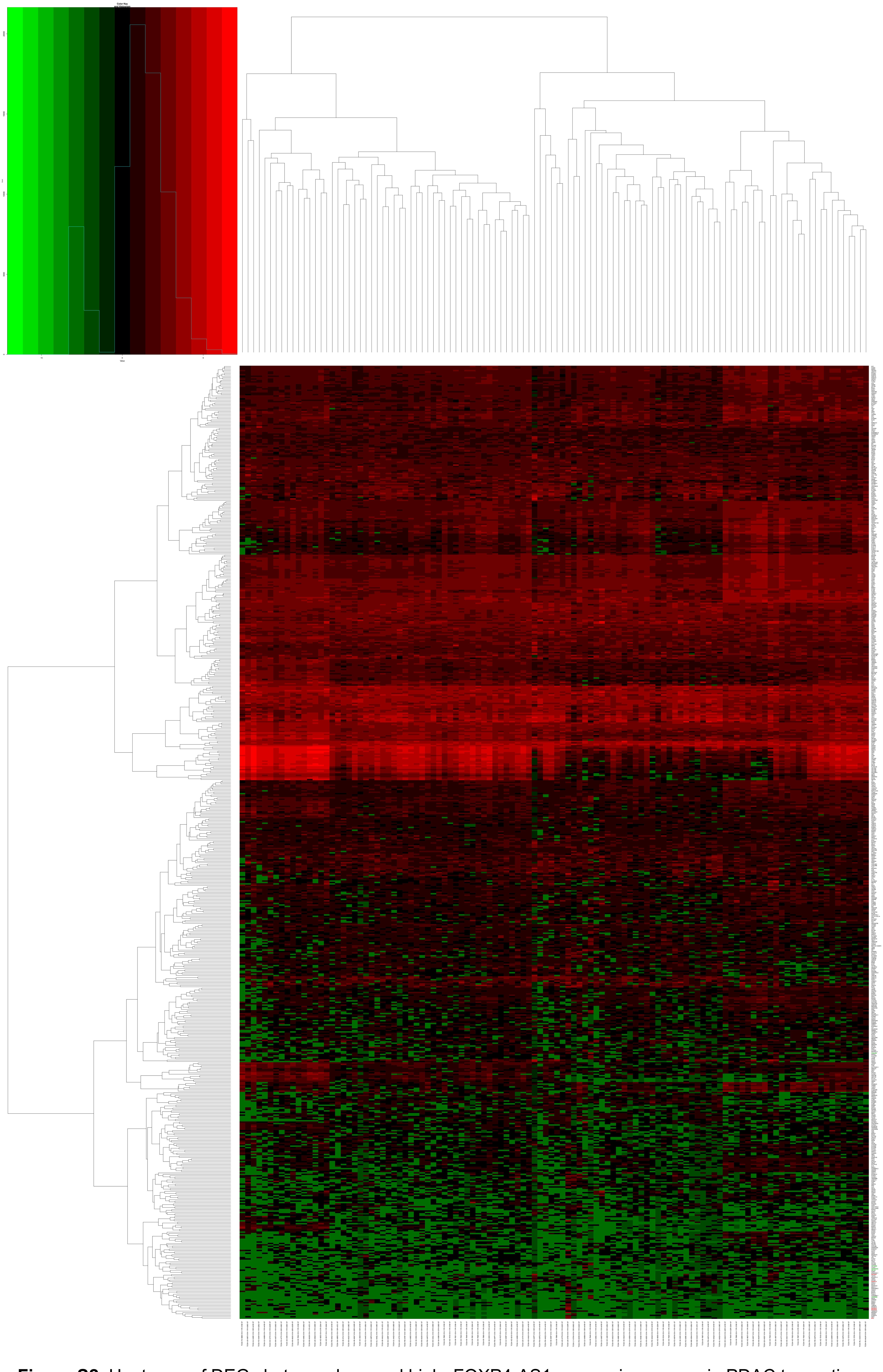

Figure S2. Heat map of DEGs between low- and high- FOXP4-AS1 expression groups in PDAC tumor tissues.
